# The first complete chloroplast sequence of a major tropical timber tree in the Meranti family: *Vatica odorata* (Dipterocarpaceae)

**DOI:** 10.1080/23802359.2016.1275837

**Published:** 2017-02-02

**Authors:** Tijana Cvetković, Damien Daniel Hinsinger, Joeri Sergej Strijk

**Affiliations:** a Plant Ecophysiology & Evolution Group, Guangxi Key Laboratory of Forest Ecology and Conservation (under state evaluation status), College of Forestry, Nanning, Guangxi, China;; b State Key Laboratory for Conservation and Utilization of Subtropical Agro-bioresources, College of Forestry, Guangxi University, Nanning, Guangxi, PR China

**Keywords:** Dipterocarpaceae, complete chloroplast genome, *Vatica odorata*, malvales, Tropical hardwood

## Abstract

Dipterocarpaceae are one of the economically most important native tree families for timber production in tropical Asia. We report the complete chloroplast genome of *Vatica odorata* (Griff.) Symington, the first in the family Dipterocarpaceae. The chloroplast genome was 151,465 bp in length, with a large single-copy (LSC) region of 83,538 bp and a small single-copy (SSC) region of 20,095 bp, separated by two inverted repeat (IRs) regions of 23,916 bp. It contained 126 genes, including 90 coding genes, 30 tRNA genes, and 8 rRNA genes. The overall GC content was 37.2%, and 43.1%, 35.2%, and 33.3% in the IRs, LSC and SSC regions, respectively. A phylogenetic tree showed *Vatica* accumulated more variation when compared with *Tilia*, and that internal relationships in Malvales need to be reassessed.

Dipterocarpaceae, comprising more than 550 species, are spread throughout tropical Asia, Africa, and South America (Gottwald & Parameswaran 1966; Ashton 1982), with their centre of diversity in Malaysia (Ashton 1988). In China, Dipterocarpaceae are represented by 5 genera and 12 species (Xi-wen et al. 2007).

Despite a number of previous studies using single or multiple marker approaches (both cp- and nDNA) for Dipterocarpaceae (Kajita et al. 1998; Gamage et al. 2006; Yulita 2013), no studies to date have generated and used complete genomes (nuclear or chloroplast).

The genus *Vatica* comprises about 65 species (Xi-wen et al. 2007). Its timber is especially valuable as it is very hard and resistant to insect attacks. However, at the current rate of forest exploitation, 65 million ha of forest are projected to be cleared for agriculture between 2012 and 2030 (FAO 2012). Here, we report the complete chloroplast sequence of *Vatica odorata* to provide genomic resources for timber identification and quality improvement.

Genomic DNA of one individual of *Vatica odorata* was extracted from fresh leaves collected in XTBG Botanical Garden (22°35′24″N, 99°30′01″E Yunnan, China – voucher STRIJK_1594 deposited in the herbarium of the College of Forestry of Guangxi University, Nanning, China), using a SDS protocol (modified from Healey et al. 2014). Library construction and sequencing were performed by Novogene (Beijing, China), following Illumina HiSeq2500 system manufacturer instructions. *De novo* assembly of the cp genome was conducted using org.asm v0.2.05 (ORG.ASM 2016) and annotation using cpGAVAS (Liu et al. 2016).

The complete cp genome of *Vatica odorata* (GenBank accession KX966283) was 151,465 bp in length, with a large single-copy (LSC) region of 83,538 bp and a small single-copy (SSC) region of 20,095 bp, separated by two inverted repeat (IRs) regions of 23,916 bp. The genome contained 126 genes, including 90 coding genes, 30 tRNA genes, and 8 rRNA genes. The overall GC content of the cp genome was 37.2% and 43.1% in IRs, which was greater than LSC (35.2%) and SSC (33.3%) regions.

The chloroplast genome of *Vatica odorata* is the first to be published for the family and to confirm the phylogenetic position within the broader Malvales order, we reconstructed phylogenetic relationships using other plastome sequences available in GenBank using PHYML (Guindon et al. 2005). Phylogenetic tree shows the particularly long branch of *Vatica*, as opposed to *Tilia*, and the short branches underlying the nodes of the preceding clades (Figure 1). However, Malvaceae in its current circumscription are not monophyletic. Our results also suggest that the merger of Tiliaceae with other Malvaceae (*sensu* Chase & Reveal 2009) does not aid in resolving the phylogenetic quagmire in the family.

**Figure 1.**
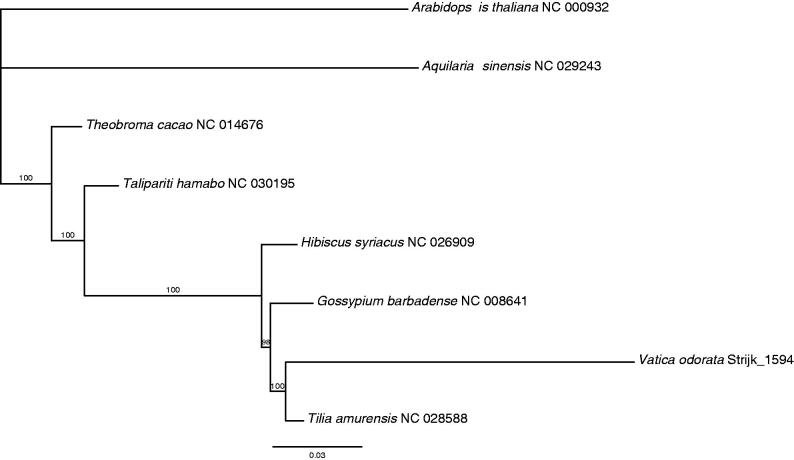
ML phylogenetic tree of the six Malvales in available chloroplast sequences in GenBank, plus the chloroplast sequence of *Vatica odorata*. The tree is rooted with the Brassicales (*Arabidopsis thaliana*). Bootstraps (100 replicates) are shown at the nodes. Scale in substitution per site.

This is the first study to report the complete chloroplast genome of a Dipterocarpaceae species and will be of great interest in further studies on genomic diversity, origin, and evolution of Dipterocarpaceae.
